# The potential use and experimental validation of genomic instability-related lncRNA in pancreatic carcinoma

**DOI:** 10.1097/MD.0000000000035300

**Published:** 2023-09-15

**Authors:** Xiuli Xia, Shushan Zhao, Xiaoming Song, Mengyue Zhang, Xinying Zhu, Changjuan Li, Wenting Chen, Dongqiang Zhao

**Affiliations:** a Department of Gastroenterology, The Second Hospital of Hebei Medical University, Shijiazhuang, China; b Department of Gastroenterology, Handan Central Hospital, Handan, China; c Department of Gastroenterology, The Third Hospital of Hebei Medical University, Shijiazhuang, China; d Department of Gastroenterology, The First Hospital of Handan, Handan, China; e Digestive Endoscopy Center, The First Affiliated Hospital of Hebei North. University, Zhangjiakou, China.

**Keywords:** genomic instability, immunotherapy, pancreatic adenocarcinoma (PAAD), the cancer genome atlas (TCGA), tumor microenvironment (TME)

## Abstract

This study explored the potential role of long noncoding RNA (lncRNAs) associated with genomic instability in the diagnosis and treatment of pancreatic adenocarcinoma (PAAD). Transcriptome and single-nucleotide variation data of PAAD samples were downloaded from the cancer genome atlas database to explore genomic instability-associated lncRNAs. We constructed a genomic instability-associated lncRNA prognostic signature. Then gene ontology and Kyoto encyclopedia of genes and genomes enrichment analyses were used to explore the physiological role of lncRNAs involved in genomic instability. Tumor microenvironments, immunotherapy response, immune cell infiltration, immune checkpoint, and drug sensitivity were compared between high-risk and low-risk groups. In vitro experiments were performed for external validation. Six lncRNAs associated with genomic instability were identified, capable of predicting the prognosis of PAAD. Patients were assigned to low-risk or high-risk groups using these biomarkers, with better or worse prognosis, respectively. The tumor immune score, immune cell infiltration, and efficacy of immunotherapy were worse in the high-risk group. A drug sensitivity analysis revealed the high- and low-risk groups had different half-maximal inhibitory concentrations. The expression of cancer susceptibility candidate 8 was significantly higher in tumor tissues than in normal tissues, while the expression of LYPLAL1-AS1 exhibited an opposite pattern. They may be potential diagnostic or prognostic biomarkers for patients with pancreatic cancer. Genomic instability-associated lncRNAs were explored in this study and predicted the prognosis of PAAD and stratified patients risk in PAAD. These lncRNAs also predicted the efficacy of immunotherapy and potential therapeutic targets in PAAD.

## 1. Introduction

Pancreatic adenocarcinoma (PAAD) ranks 7th as the cause of cancer-related deaths. It is a malignancy with poor prognosis and high mortality and it cannot be effectively screened, most patients suffer locally advanced or metastatic disease at diagnosis.^[1]^ The 5-year survival rate for patients with metastatic pancreatic cancer is about 3%.^[2]^ PAAD has an increasing incidence rate but its survival rate roughly improved, which may be attributed to difficulties in early detection, low probability of radical resection, and resistance to chemotherapy and radiotherapy.^[3]^ Recently, significant progress has been made in tumor diagnosis and targeted therapy. However, effective biomarkers for early diagnosis and prognosis of PAAD, a highly heterogeneous disease, have not yet been identified. Targeted therapy and immunotherapy have not yet played an active role in pancreatic cancer.^[4,5]^ Advances in these areas can improve the individualized treatment of patients and lay a foundation for further research on the underlying molecular mechanisms of PAAD.

Recently, much attention has been paid to long noncoding RNAs (lncRNAs), which are more than 200 nucleotides in length and have no protein-coding capability.^[6]^ Data from the Human Genome Project have suggested that protein-coding genes account for <2% of total gene sequences.^[7]^ lncRNAs can regulate transcription and have been proven to participate in the development and progression of almost all types of tumors.^[8]^ Expression of lncRNAs has been discovered in PAAD tissues and cell lines using microarray and quantitative reverse transcription (qRT)-PCR. For instance, MACC1-AS1, an lncRNA frequently expressed in PAAD tissues, is particularly overexpressed in patients with poor survival.^[9]^ A previous study elucidated the involvement of lncRNAs in the development and treatment of PAAD as carcinogenic factors or tumor suppressors, thus highlighting lncRNAs as potential diagnostic and therapeutic targets.^[10–13]^

It is now generally believed that genomic instability may have greater diagnostic potential for early detection of cancer.^[14]^ Recent studies have found that molecular subtypes of pancreatic cancer are associated with specific copy number abnormalities of mutated genes such as Kirsten rat sarcoma viral oncogene homolog (KRAS).^[15]^ KRAS is a member of the RAS family encoding small GTPases and the mutation exists in approximately 85% of pancreatic ductal adenocarcinoma and in early-stage PAAD. It is detected in 25% and 38% of pancreatic intraepithelial neoplasia-1A and pancreatic intraepithelial neoplasia-1B cases. Thus, extensive evidence suggests that KRAS mutation may be an early and initiating event in human PAAD.^[16]^ Studies have shown that genomic instability-associated lncRNAs are present in several types of tumors, such as gastric cancer,^[17]^ but have rarely been reported in PAAD, a tumor with low mutation frequency. More specifically, lncRNAs associated with genomic instability and their value in predicting prognosis and the response to immunotherapy in patients with PAAD have not been widely investigated.

Immunotherapy modalities, particularly immune checkpoint inhibitors and GM-CSF cell vaccines (GVAX), have revolutionized cancer treatment in recent years. However, immunotherapy has not been successful against pancreatic cancer,^[18]^ partly due to the complex and heterogeneous tumor microenvironment (TME), which generally consists of a fibroproliferative stroma depleted of immune cells.^[19]^ Genomic instability is closely related to tumor immunotherapy because genomic instability produces tumor neoantigens, which are recognized by antigen-presenting cells. By presenting tumor-associated antigens, dendritic cells activate T lymphocytes to differentiate into cytotoxic T lymphocytes, which enter tumor tissues and kill tumor cells. Necrotic tumor cells then release tumor neoantigens and participate in the next round of tumor immunization.^[20]^ Thus, we studied the association of lncRNAs with genomic instability and response to immunotherapy. A better understanding of the immunotherapy and tumor microenvironment of PAAD is crucial to improve individualized treatment strategies. In particular, the relationship between genomic instability and prognosis of pancreatic cancer can help identify new prognostic biomarkers and therapeutic targets.

## 2. Methods

### 2.1. Data collection

**Figure 1.**
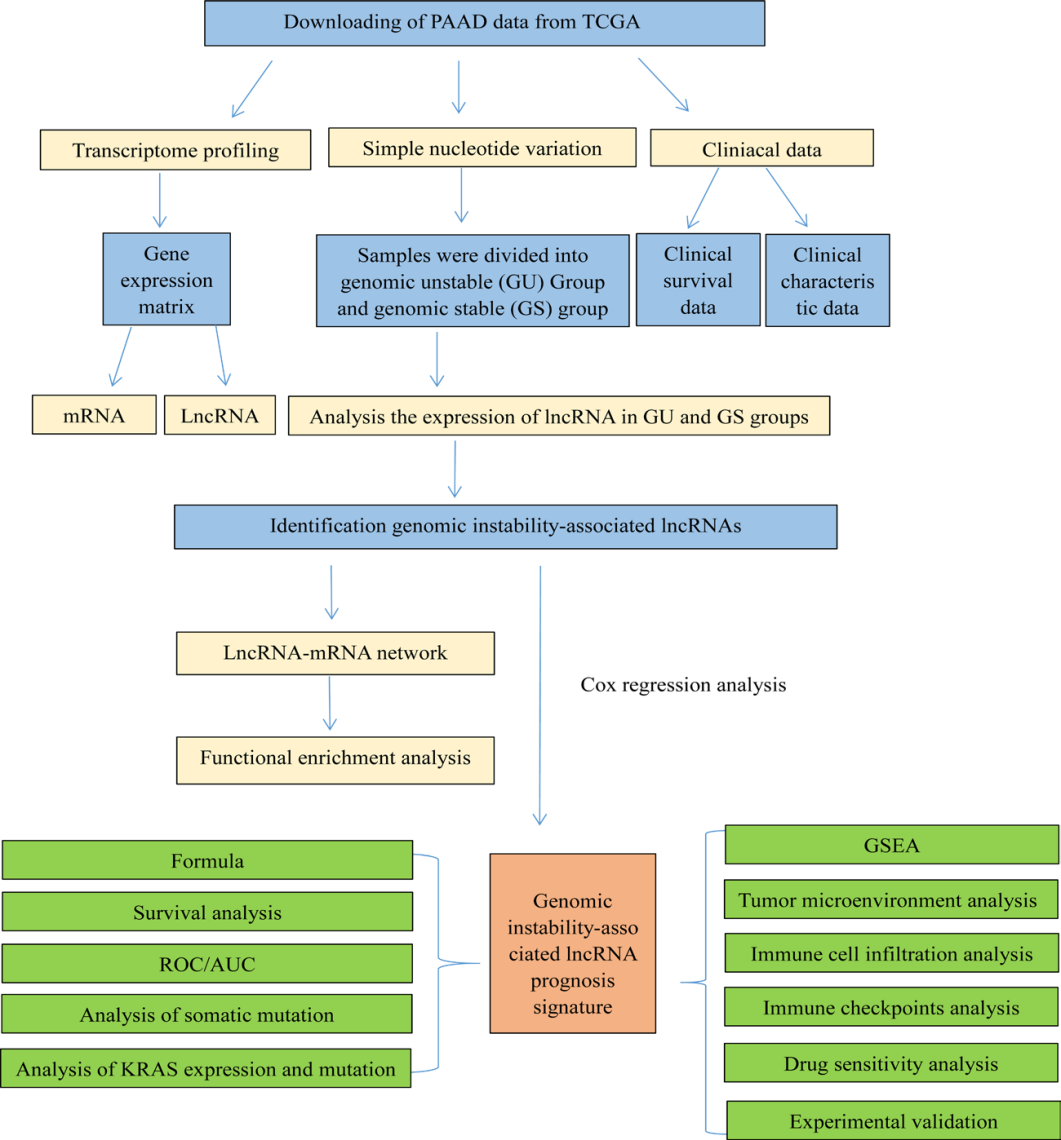
Workflow diagram.

Transcriptome data (n = 178), single-nucleotide variation data (n = 158) and clinical data (n = 185) of patients with PAAD were downloaded from the cancer genome atlas database (TCGA) database. (https://portal.gdc.cancer.gov/). Patients (excluding those with survival time < 30 days) were randomly assigned to a training cohort and testing cohort. A prognosis signature was subsequently constructed using data in the training cohort and validated using data in the testing cohort. The workflow diagram is shown in Figure 1.

### 2.2. Analysis of single-nucleotide variation data and identification of genomic instability-associated lncRNAs

The cumulative number of somatic mutations in each patient was sorted and ranked in descending order. Those exhibiting the highest 25% were assigned to a genomic unstable (GU) group, and those with the lowest 25% were assigned to a genomic stable (GS) group. The expression levels of lncRNAs in the 2 groups were differentially analyzed using R package “limma.” LncRNAs with |log2 (fold change)| > 1 and false discovery rate < 0.05 were screened and defined as genomic instability-associated lncRNAs. In order to further study lncRNAs associated with genomic instability, using hierarchical cluster algorithm, patients with PAAD were assigned to 2 groups according to genomic instability-associated lncRNAs, which were designated GU-like group and GS-like group. Correlation analysis was then performed between mutation frequency and gene expression in the 2 groups.

### 2.3. Gene ontology (GO) and Kyoto encyclopedia of genes and genomes (KEGG) analyses

Pearson correlation coefficient was utilized to evaluate expression correlations between genomic instability-associated lncRNAs and protein-coding genes. The 10 protein-coding genes displaying the greatest correlation were used to construct an lncRNA-mRNA co-expression network by means of R package “igraph,” followed by GO and KEGG analyses using R 4.1.2 to identify the functions and pathways involved.

### 2.4. Construction of genomic instability-associated lncRNA prognosis signature

Genomic instability-associated lncRNA expression, the survival time and survival status of patients with PAAD were subjected to univariate Cox regression analysis to produce a genomic instability-associated lncRNA prognosis signature, Patients were assigned to high-risk and low-risk groups using the median risk score of the training cohort. Survival difference between high- and low-risk groups was probed by Kaplan–Meier analysis. The signature’s accuracy was evaluated by the receiver operating characteristic (ROC) curve. ROC analysis of our signature was compared with other clinical characteristics and previously discovered biomarkers. In addition, “ggpubr” and “limma” packages were utilized to analyze the correlation of somatic mutation with gene expression in high- and low-risk groups. Relevant physiological functions of the signature involved in PAAD were elucidated by gene set enrichment analysis (GSEA) and enrichment pathways in high- and low-risk groups. Analysis was conducted using R packages “plyr,” “ggplot2,” “grid” and “gridExtra.” R 4.1.2 was applied for all statistical analyses.

### 2.5. Correlation of the signature with immunotherapy and Tumor microenvironment

Spearman analysis was employed to investigate correlations between the prognostic signature and immune cells. R packages “limma” “scales” “ggplot2” “ggtext” “reshape2” “tidyverse” and “ggpubr” were utilized to incorporate predicted results obtained from multiple softwares (XCELL TIMER, QUANTISEQ, MCPCOUNTER, EPIC, CIBERSORT-ABS, and CIBERSORT) into the bubble chart. The tumor microenvironment was analyzed using ESTIMATE^[21]^ and R packages “limma” and “ggpubr,” to determine whether differences were present in stromal score, immune score and ESTIMATE score between high- and low-risk groups. Identification of immune cells could not be performed using by ESTIMATE. Thus, differences in immune cells and immune-related functions between high- and low-risk groups were evaluated using single sample gene set enrichment analysis ^[22]^ and R packages “GSVA” “limma” “GSEA Base” “ggpubr” and “reshape2.” In addition, the difference in immune checkpoint-related genes between high- and low-risk groups was analyzed by means of R packages “limma,” “reshape2,” “ggplot2” and “ggpubr.”

### 2.6. Drug sensitivity

The relationship between the prognostic signature and drug sensitivity was explored by assessing differences of half-maximal inhibitory concentrations (IC_50_) between high- and low-risk patient groups. Analysis was performed using R packages “pRRophetic,” “limma,” “ggpubr” and “ggplot2.”

### 2.7. Expression analysis of genomic instability-associated lncRNAs using the UCSC Xena database

TCGA database has a small number of normal tissue samples, while the UCSC Xena database comprises public data from TCGA, Genotype-Tissue Expression, International Cancer Genome Consortium, and other databases. We combined tumor tissue samples and normal tissue samples from the UCSC Xena database. In total, 178 PAAD samples and 171 normal samples were collected. The expression levels of each lncRNA in our signature in PAAD and normal tissues were analyzed.

### 2.8. Validation of the expression level of genomic instability-associated lncRNAs in tissue specimens by qRT-PCR

Nineteen pairs of PAAD and adjacent normal tissue samples were collected from Handan Central Hospital. All tissues were immediately placed in liquid nitrogen and stored at −80°C. All patients had not received radiotherapy or chemotherapy, and pathological tissues were confirmed by experienced pathologists. The expression levels of cancer susceptibility candidate 8 (CASC8) and LYPLAL1-AS1 were detected by (qRT) -PCR. The 2 ^−ΔΔCt^ method was used to calculate the relative expression levels. GAPDH was selected as the internal reference. The primers are listed in Table 1.

**Table 1:** Primers used in qRT-PCR.

Primer	Sequence
LYPLAL1-AS1-F	CGCCAACCTTGGATGTCTCAGTC
LYPLAL1-AS1-R	CCTCCACACAATCTGTCCCGAAAG
CASC8-F	TGTCCTCCTCCTTGCTGGTATACAC
CASC8-R	TCACCTGCTTCAGTGCCATTGTC
GAPDH-F	CAGGAGGCATTGCTGATGAT
GAPDH-R	GAAGGCTGGGGCTCATTT

CASC8 = cancer susceptibility candidate 8, qRT = quantitative reverse transcription.

### 2.9. Statistical analysis

R 4.1.2 was adopted for statistical analysis throughout this study. Kruskal Wallis test and 1-way analysis of variance were applied to nonparametric and parametric data. Spearman correlation was employed to analyze the correlations between 2 continuous variables.

## 3. Results

### 3.1. Identification of genomic instability-associated lncRNAs

**Figure 2.**
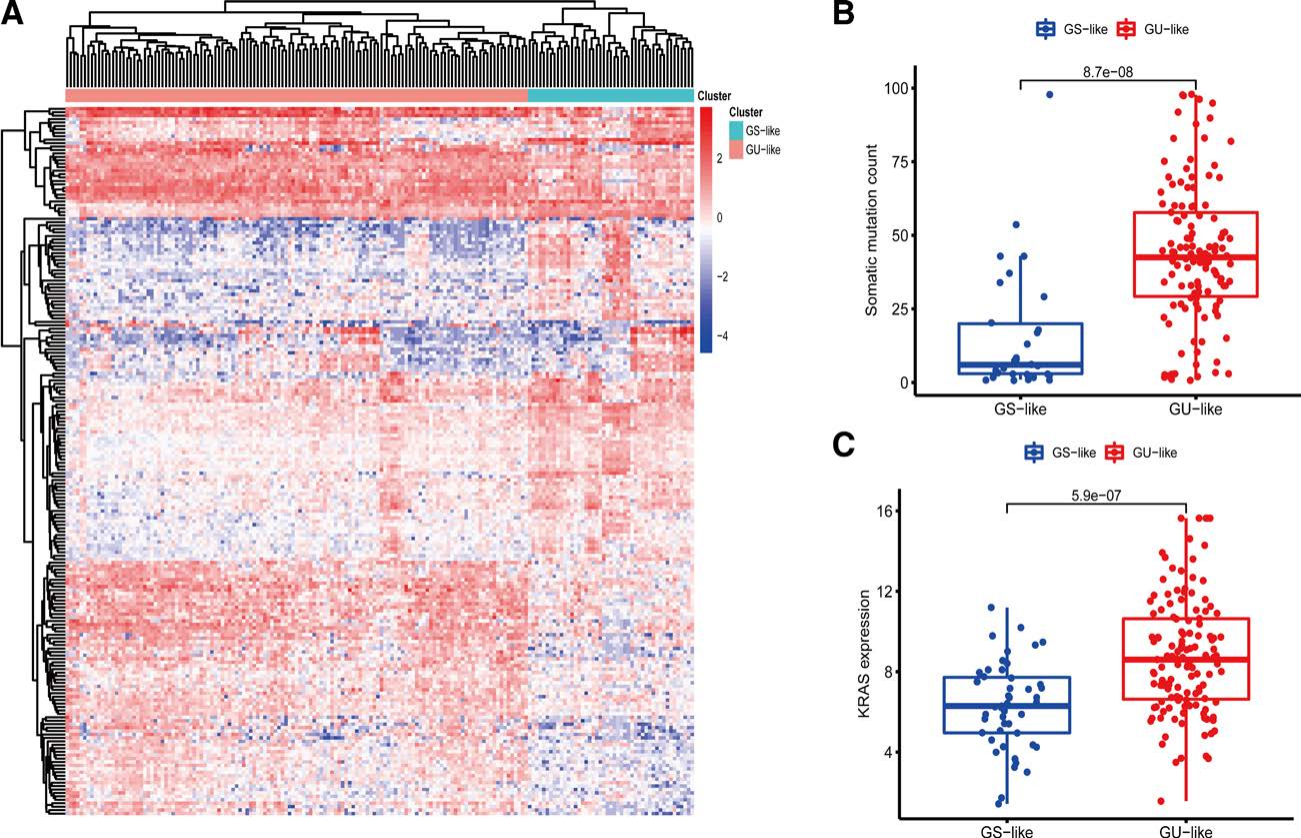
Heatmap of genomic instability-associated lncRNAs and mutation-related analysis. (A) Heatmap of genomic instability-associated lncRNAs. Red color indicates GU-like group and blue color indicates GS-like group. (B) Boxplot showing the mutation frequencies of GS-like and GU-like groups. Red color indicates GU-like group, and blue color indicates GS-like group. (C) Boxplot showing the expression of KRAS in GS-like and GU-like groups. Red color indicates GU-like group, and blue color indicates GS-like group. KRAS = Kirsten rat sarcoma viral oncogene homolog, lncRNAs = long noncoding RNA, GU = genomic unstable, GS = genomic stable.

The somatic mutation count in 158 PAAD patients was sorted in descending order; those ranked in the top 25% were assigned to the GU group and those in the lowest 25% were assigned to the GS group. Differentially expressed lncRNAs were screened out from GU and GS groups with |log2 (fold change)| > 1 and false discovery rate < 0.05 as the threshold, then differentially expressed lncRNAs were screened, of which 95 were up-regulated and 111 were down-regulated in the GU group. The lncRNAs were subjected to hierarchical cluster analysis, and patients were categorized into GS-like (n = 47) and GU-like groups (n = 131) (Fig. 2A).

In order to identify and define the distinguishing features of patient classification, the mutation frequencies of GS-like and GU-like groups were analyzed, indicating that the mutation frequency of the GU-like group was higher than that of the GS-like group (*P* = 8.7*e*–08) (Fig. 2B). KRAS gene mutation is considered to be an important driver of pancreatic cancer. Expression of KRAS was compared in GS-like and GU-like groups, revealing statistically significant increased expression of KRAS in the GU-like group (*P* = 5.9*e*–07) (Fig. 2C).

### 3.2. GO and KEGG enrichment analyses

**Figure 3.**
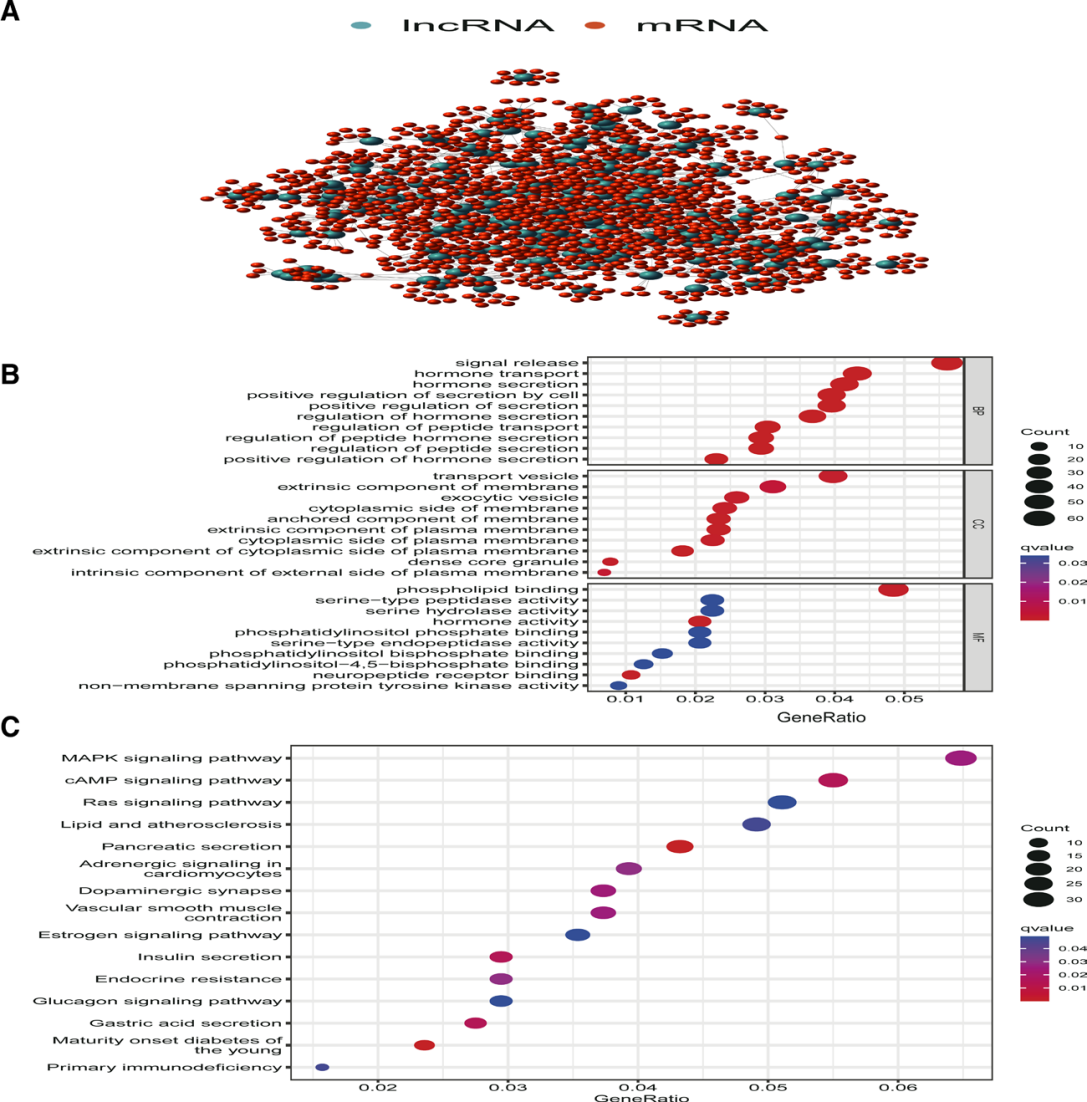
LncRNA-mRNA network and enrichment analysis. (A) LncRNA-mRNA network. Blue dots: genomic instability-associated lncRNAs. Red dots: the top 10 corresponding mRNAs. (B) Bubble diagram of GO enrichment analysis. (C) Bubble diagram of KEGG pathway enrichment analysis. GO = gene ontology, KEGG = Kyoto encyclopedia of genes and genomes, lncRNAs = long noncoding RNA.

In order to explore the functions of the genomic instability-associated lncRNAs, the corresponding mRNAs were identified and the 10 protein-coding genes with highest correlation were used to construct an lncRNA-mRNA network (Fig. 3A). Subsequent, GO enrichment analysis indicated the lncRNAs-related mRNAs were involved in signal release (biological process), phospholipid binding (molecular function) and transport vesicle (cellular component) (Fig. 3B). KEGG pathway enrichment analysis indicated that these genes were prevalent in pancreatic secretion and maturity onset diabetes of the young (Fig. 3C).

### 3.3. Construction of genomic instability-associated lncRNAs prognostic signature

**Figure 4.**
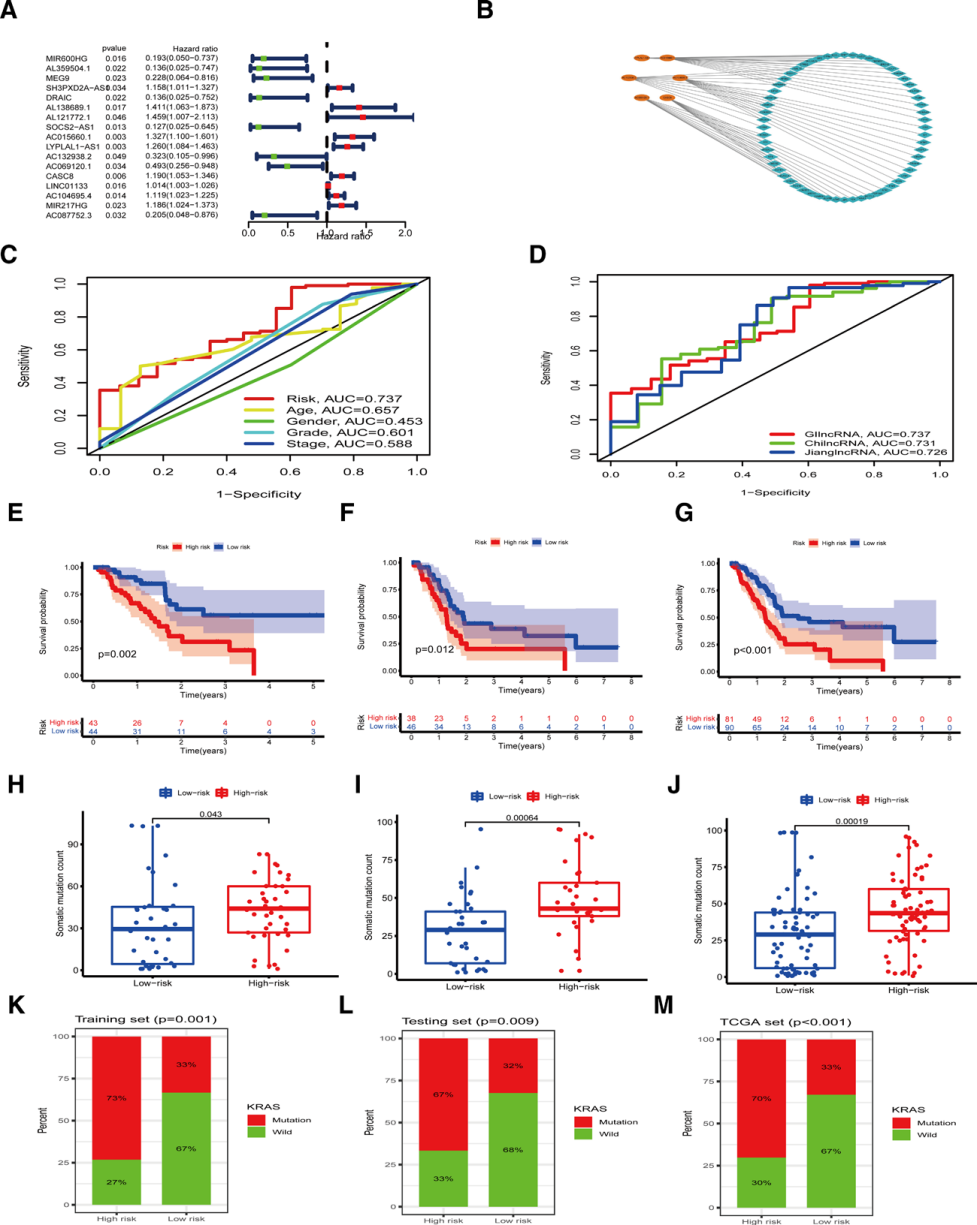
Forest plot, prognostic value analysis of genomic instability-related lncRNAs. (A) Forest plot showing univariate COX regression analysis of prognosis-related lncRNAs in PAAD. (B) The network of genomic instability-related lncRNAs signature and their closely related mRNAs. Orange color indicates the lncRNAs that constitute the signature, and the blue diamonds represent their corresponding mRNAs. (C) The ROC analysis of the lncRNA signature and other clinical characteristics. (D) ROC curve comparison of genomic instability-related lncRNA signatures with previously identified signatures. (E–G) The signature predicted Kaplan–Meier survival curves of patients in the high- and low-risk groups in training cohort, testing cohort and all cohorts. (H–J) Somatic mutation counts in the high-risk and low-risk groups of the training cohort, testing cohort and all cohorts. (K–M) The frequency of KRAS gene mutation in high-risk and low-risk groups in the training cohort, testing cohort and all cohorts. (E, H, K) Training cohort. (F, I, L) Testing cohort. (G, J, M) All cohorts. AUC = area under the curve, KRAS = Kirsten rat sarcoma viral oncogene homolog, lncRNAs = long noncoding RNA, PAAD = pancreatic adenocarcinoma, ROC = receiver operating characteristic.

The expression of genomic instability-associated lncRNAs in each patient with PAAD was correlated with survival time and 17 prognosis-related lncRNAs were identified by univariate Cox regression analysis (*P* < .05) (Fig. 4A). Six lncRNAs (AC015660.1, LYPLAL1-AS1, AC132938.2, AC069120.1, CASC8, and AC104695.4) with independent prognostic value were selected for construction of the predictive signature (Fig. 4B). The signature score was calculated by the sum of the expression level of each lncRNA multiplied by its coefficient. Patients were randomly divided into the training cohort and testing cohort, with no significant difference in clinical features between the 2 groups (*P* < *.05*) (Table 2). Patients were then divided into the high-risk group and low-risk group in both cohorts according to the median risk score of the training cohort. The ROC analysis of the lncRNA biomarkers and clinical characteristics showed the area under the curve value of risk score was higher than age, gender, tumor grade and stage (Fig. 4C). Comparing the ROC curve of genomic instability-related lncRNAs with previously identified biomarkers indicated that our biomarkers had a higher prognostic value than previously identified lncRNAs signatures (Fig. 4D).^[23,24]^ Kaplan–Meier analysis confirmed that prognosis was improved in the low-risk group compared with the high-risk group in the training, testing and all cohorts (*P* < .05) (Fig. 4E–G). We also analyzed the correlations between the prognostic signature and gene mutation. Results showed that the high-risk patient group exhibited a higher somatic mutation frequency than the low-risk group in the training, testing and all cohorts (*P* < .05) (Fig. 4H–J). Further analysis showed that the mutation rate of KRAS gene was statistically higher in the high-risk group than in the low-risk group in all cohorts (Fig. 4K–M).

**Table 2:** Clinical characteristics of patients.

Covariates	Type	Total	Test	Train	*P* value
Age	<=60	56 (32.94%)	29 (34.94%)	27 (31.03%)	.7052
Age	>60	114 (67.06%)	54 (65.06%)	60 (68.97%)	
Gender	FEMALE	77 (45.29%)	38 (45.78%)	39 (44.83%)	1
Gender	MALE	93 (54.71%)	45 (54.22%)	48 (55.17%)	
Grade	G1–2	119 (70%)	57 (68.67%)	62 (71.26%)	.661
Grade	G3–4	49 (28.82%)	26 (31.33%)	23 (26.44%)	
Grade	Unknown	2 (1.18%)	0 (0%)	2 (2.3%)	
Stage	Stage I–II	160 (94.12%)	82 (98.8%)	78 (89.66%)	.1264
Stage	Stage III–IV	7 (4.12%)	1 (1.2%)	6 (6.9%)	
Stage	Unknown	3 (1.76%)	0 (0%)	3 (3.45%)	
T	T1–2	27 (15.88%)	15 (18.07%)	12 (13.79%)	.6258
T	T3–4	141 (82.94%)	68 (81.93%)	73 (83.91%)	
T	Unknown	2 (1.18%)	0 (0%)	2 (2.3%)	
M	M0	77 (45.29%)	35 (42.17%)	42 (48.28%)	.2035
M	M1	4 (2.35%)	0 (0%)	4 (4.6%)	
M	Unknown	89 (52.35%)	48 (57.83%)	41 (47.13%)	
N	N0	47 (27.65%)	19 (22.89%)	28 (32.18%)	.2366
N	N1–3	119 (70%)	62 (74.7%)	57 (65.52%)	
N	Unknown	4 (2.35%)	2 (2.41%)	2 (2.3%)	

### 3.4. Analysis of prognostic value of the signature

**Figure 5.**
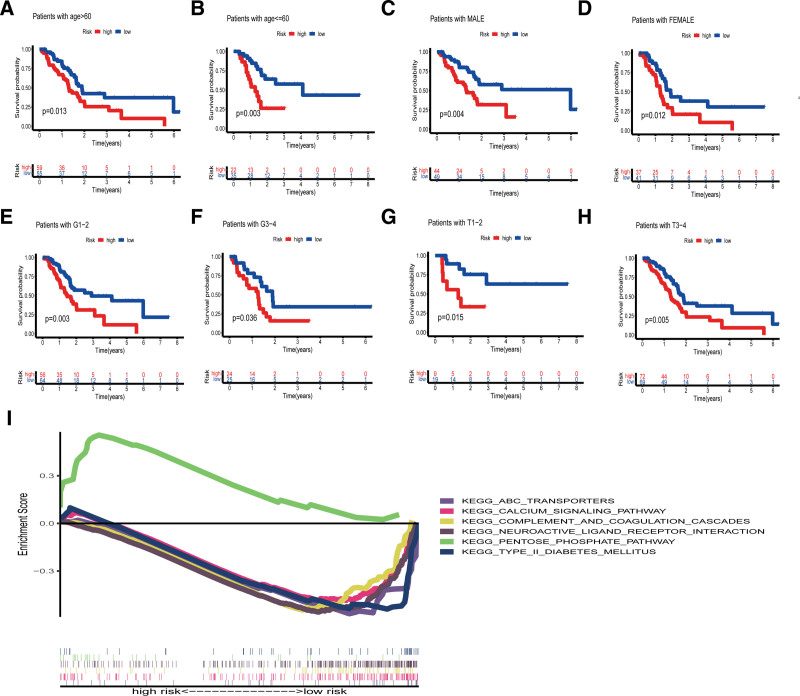
Survival curves analysis and GSEA. (A–H) Survival curves for validating the prognostic signature based on clinical characteristics: age, sex, pathological staging, and T-staging. (I) Gene Set Enrichment Analysis (GSEA): pathway with enrichment scores > 0 active in the high-risk group, while pathways with enrichment scores < 0 active in the low-risk group.

We validated the prognostic signature according to clinical traits and found that the signature was suitable for clinical characteristics of age, gender, pathological stage, and T-staging. According to our signature, each patient was assigned to a high or low-risk group based on the median risk score, and all patients were assigned by age (age > 60, age < = 60), gender (female, male), pathological stage (G1–2, G3–4) and T stage (T1–2, T3–4) showed significant differences in high- and low-risk groups (Fig. 5A–H). Cox regression analyses indicated that the signature and grade can serve as independent prognostic predictor (Table 3).The relationship between the signature and PAAD-related pathways was analyzed using GSEA. Results suggested that high-risk patients were mainly involved with the pentose phosphate pathway (PPP). Low-risk patients in comparison, evidenced involvement of ABC transporters, calcium signaling pathway, Type II diabetes mellitus, as well as complement and coagulation cascades (Fig. 5I).

**Table 3:** Univariable and multivariable Cox regression analyses of the signature risk score and clinical characteristics.

	Univariable				Multivariable				
	Variables	HR	HR.95L	HR.95H	*P* value	HR	HR.95L	HR.95H	*P* value
	Age	1.0246	0.9920	1.0582	.1399				
	Gender	0.7154	0.3770	1.3575	.3055				
Training	Grade	1.0349	0.6406	1.6720	.8882				
	Stage	1.2513	0.7596	2.0614	.3785				
	Risk score	1.1438	1.0868	1.2039	2.62E-07	1.1438	1.0868	1.2039	2.62E-07
	Age	1.0279	0.9997	1.0568	.0520				
	Gender	1.0230	0.5920	1.7678	.9349				
Testing	Grade	1.6531	1.1469	2.3827	.0070	1.5750	1.1016	2.2518	.0127
	Stage	2.3670	0.9596	5.8384	.0613				
	Risk score	1.0536	1.0130	1.0960	.0092	1.0495	1.0050	1.0960	.0285

### 3.5. Correlation of the signature with immunotherapy and TME

**Figure 6.**
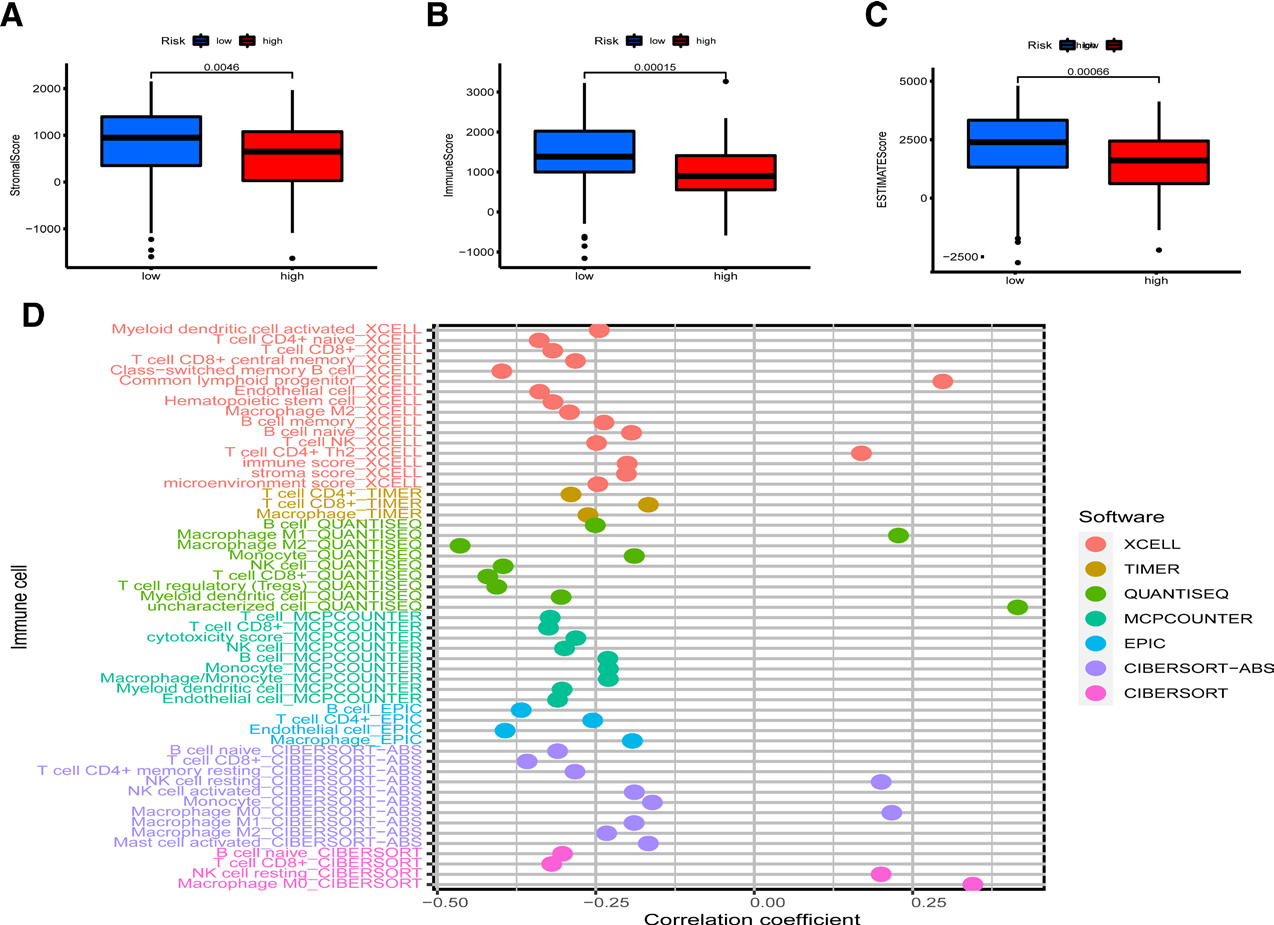
Correlation of the genomic instability-related lncRNAs signature with TME. (A–C) Stromal score, Immune score, ESTIMATE score in low-and high-risk groups (*P* < .05). (D) Bubble chart of immune cell infiltration: immune cells with a correlation coefficient > 0 are positively associated with risk score. Immune cells with a correlation coefficient < 0 are negatively associated with risk score. lncRNAs = long noncoding RNA, TME = tumor microenvironment.

**Figure 7.**
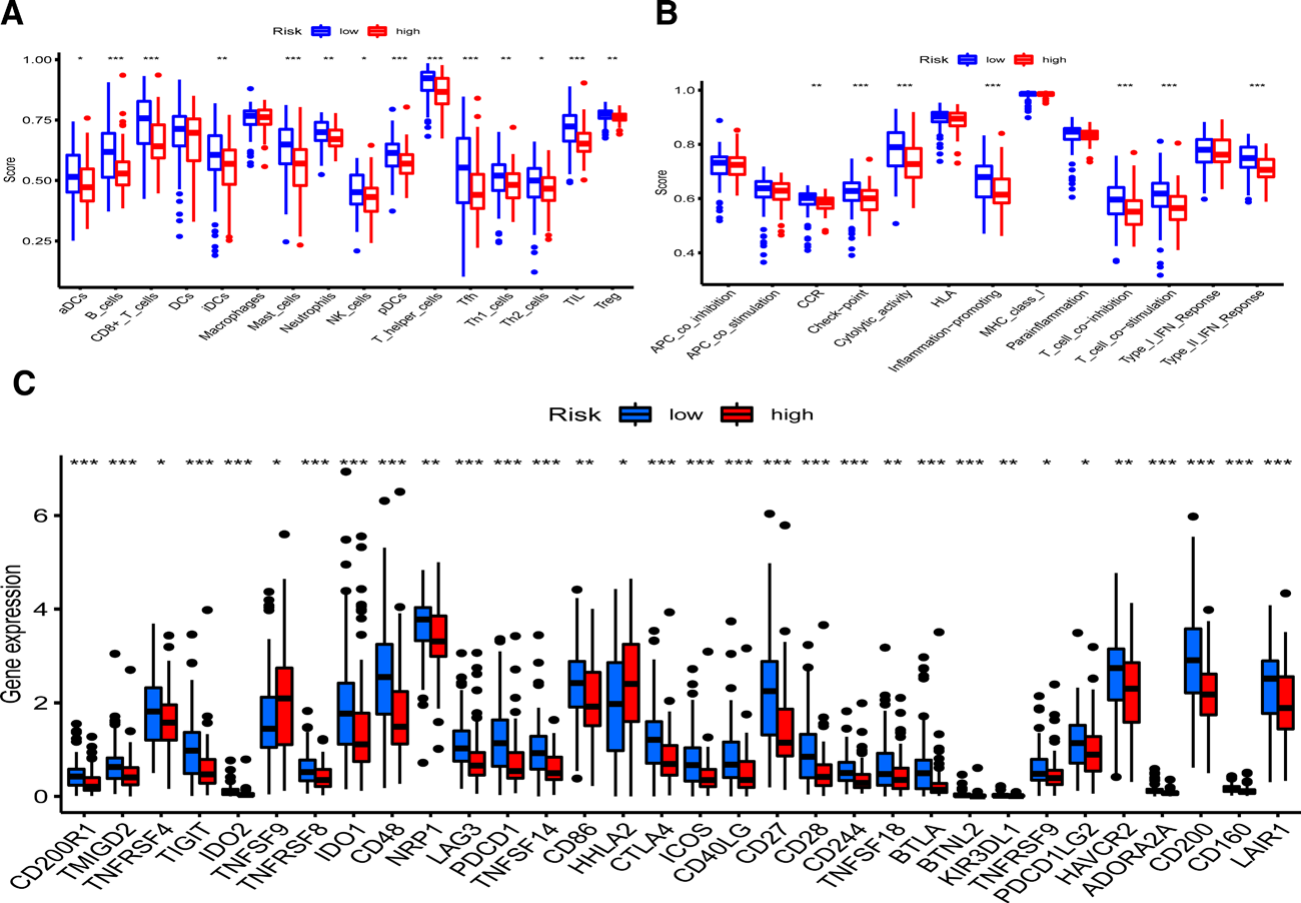
Immune-related analysis of different risk groups. (A) Differences in immune cell fractions between high- and low-risk groups. The Y-axis represents the immune cells fraction, and the X-axis represents immune cells. (B) Comparison of immune-related functions in high- and low-risk groups. The Y-axis represents the immune-related functions fraction, and the X-axis represents different immune-related functions. (C) Comparison of the expression of immune checkpoint-related genes in the high-risk and low-risk groups. The Y-axis represents the expression level, and the X-axis represents immune checkpoint-related genes. (*:*P* < .05, **:*P* < .01, ***:*P* < .001.).

The tumor microenvironment was analyzed using ESTIMATE to calculate the stromal score, immune score and ESTIMATE score for each patient with PAAD. Results showed that the high-risk group had lower stromal score, immune score, and ESTIMATE scores than the low-risk group (*P* < .001) (Fig. 6A–C). Correlation of the signature riskscore with immune cell types revealed that the vast majority of immune cells were negatively correlated with signature riskscore (Fig. 6D). Correlation of the prognostic signature with 16 types of immune cells and thirteen immune-related functions was investigated using single sample gene set enrichment analysis. The results indicated that lower infiltration of immune cells was found in the high-risk group, suggesting that immune cell infiltration was negatively correlated with our signature risk score. These findings were consistent with our previous ESTIMATE analysis and immune cell infiltration analysis. Immune-related function analysis showed that the low-risk group had a stronger immune response (Fig. 7A, B). Further analysis suggested that there were differences in the expression of all immune checkpoint-related genes between the high- and low-risk groups. Among them, TNFSF9 and HHLA2 showed higher expression in the high-risk group. (Fig.7C).

### 3.6. Drugs sensitivity analysis

**Figure 8.**
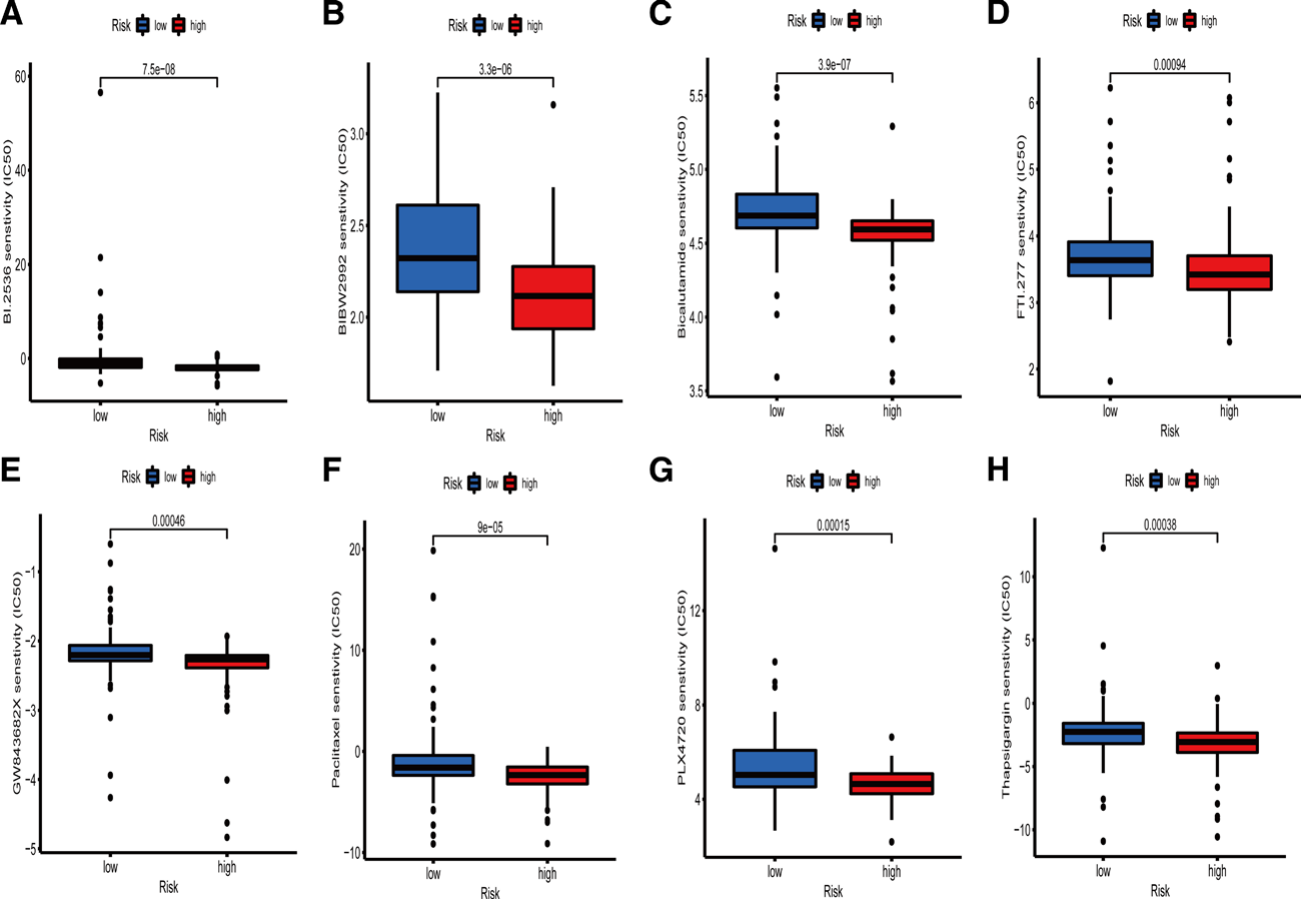
Drugs sensitivity in two risk groups. (A) BI.2536. (B) BIBW2992. (C) Bicalutamide. (D) FTI.277. (E) GW843682X. (F) Paclitaxel. (F) PLX4720. (H) Thapsigargin.

Many patients with advanced pancreatic cancer are currently being treated with drugs. We further analyzed the correlation between our signature and IC_50_. The “sensitivity analysis” of more than 130 drugs, including conventional chemotherapeutic agents and immune checkpoint inhibitors, was carried out on the high- and low-risk patient groups using the signature. Twenty-two drugs were found to exhibit different IC_50_ values between 2 risk groups. Drugs displaying lower IC_50_ in the high-risk group (*P* < .001) included BI.2536, BIBW2992, Bicalutamide, FTI.277, GW843682X, Paclitaxel, PLX4720, and Thapsigargin (Fig. 8A–H). The results shed light on PAAD patients in high-risk group could benefit from these drugs, suggesting that our signature could be a potential tool for predicting drug susceptibility.

### 3.7. qRT-PCR validation of CASC8 and LYPLAL1-AS1 expression in Chinese population.

**Figure 9.**
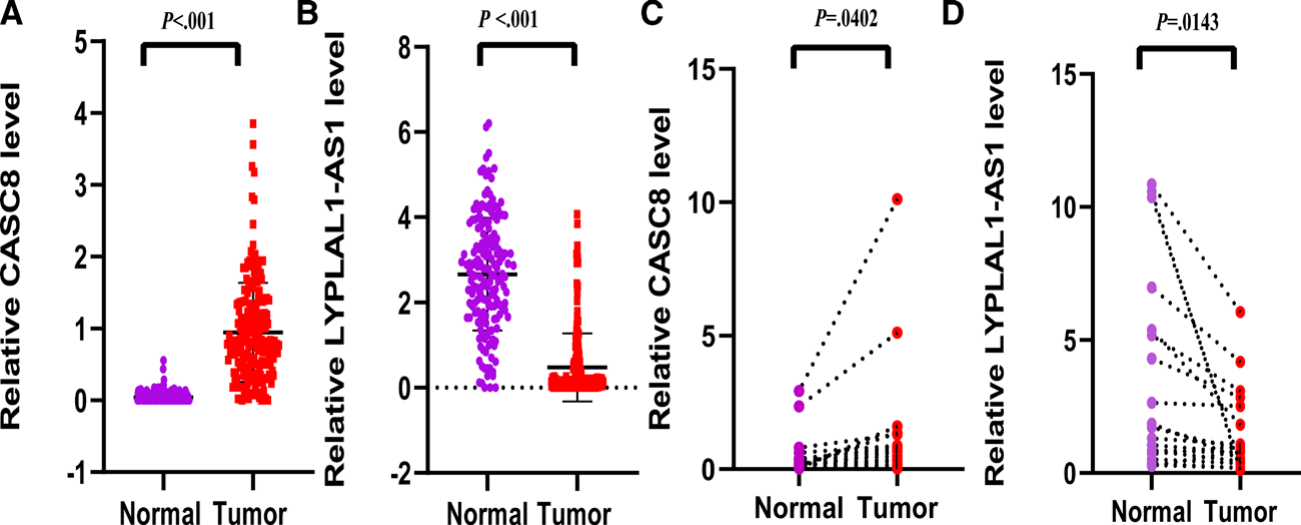
Expression analysis of CASC8 and LYPLAL1-AS1 in PAAD patients. (A, B) Relative expression level of CASC8 and LYPLAL1-AS1 in pancreatic cancer and normal pancreatic tissues based on TCGA data. (C, D) qRT-PCR verified the expression of CASC8 and LYPLAL1-AS1 in pancreatic and adjacent normal tissues of Chinese patients. CASC8 = cancer susceptibility candidate 8, PAAD = pancreatic adenocarcinoma, TCGA = the cancer genome atlas database, qRT = quantitative reverse transcription.

We used data from 178 PAAD samples and 171 normal samples downloaded from the UCSC Xena database. CASC8 and LYPLAL1-AS1 were differentially expressed in pancreatic cancer tissues and normal pancreatic tissues. The expression of CASC8 was significantly increased in tumor tissues (*P* < .001), and the expression of LYPLAL1-AS1 was significantly higher in normal tissues (*P* < .001), indicating that LYPLAL1-AS1 is a protective gene (Fig. 9A–B). To further validate the feasibility of prognostic features, qRT-PCR assay was performed to determine the expression levels of CASC8 and LYPLAL1-AS1 in 19 Chinese pancreatic tissue samples. The results showed that the expression level of CASC8 in tumor tissues was significantly higher than that in adjacent normal tissues (*P =* .0402), while the expression of LYPLAL1-AS1 showed an opposite pattern (*P =* .0143) (Fig. 9C–D). CASC8 expression was elevated in 17 of 19 pancreatic cancer samples, of which 7 were moderately differentiated and 10 were moderately-poorly differentiated according to AJCC Cancer Staging Manual/Edition 8th. CASC8 was lowly expressed in 2 tumor tissues, 1 sample was moderately differentiated (cancer stage III [T2N2]) and 1 sample was poorly differentiated (cancer stage IB [T2N0]).

## 4. Discussion

Pancreatic cancer is one of the most malignant tumors with a poor prognosis. Due to the lack of appropriate screening and diagnostic methods, difficult biopsy, rapid tumor progression, and the low response rate to treatment, the incidence and mortality of pancreatic cancer are almost the same. Therefore, it is necessary to find effective biomarkers to facilitate the early diagnosis of pancreatic cancer and improve its response to treatment.

Genomic instability has been the focus of research in recent years and closely associated with the development of nearly all types of tumors. It is characterized by the occurrence of new mutations in many sporadic human cancers and happens during tumor development and metastasis.^[25]^ Cancer cells rapidly proliferate, which increases the risk of DNA replication stress and mutations in specific genomic sites. The occurrence of tumors will induce genomic instability and easily affect these specific gene sites.^[26]^ Whole-exome sequencing of PAAD specimens showed recurrent somatic mutations of KRAS and TP53.^[27]^ KRAS mutation not only affects the cell proliferation and invasion of PAAD, but also leads to immunosuppression.^[28]^ Although lncRNAs have no protein-coding function, they can regulate transcription and affect the development, progression, and drug resistance of cancer.^[29–31]^ Several studies have been conducted to shed light on the role of genomic instability in tumorigenesis and tumor progression. On the other hand, lncRNAs are associated with the development, progression, treatment, and prognosis of pancreatic cancer.^[30,32–34]^ However, the role of lncRNAs in genomic instability and tumor progression is in its infancy.

We identified a new genomic instability-related signature composed of 6 lncRNAs and carried out the relevant experimental verification. Our findings can uncover the role of genomic instability-related lncRNAs in the prognosis and treatment of PAAD and improve the treatment of PAAD, According to the risk scores of signature composed of 6 lncRNAs, PAAD patients were divided into 2 groups of high-risk and low-risk. KRAS mutation frequency was remarkably higher in the high-risk group. Importantly, this association of genomic instability with KRAS mutation in PAAD has not been previously reported. KEGG pathway enrichment analysis indicated that genomic instability-associated lncRNAs were prevalent in pancreatic secretion and maturity onset diabetes of the young, which may be related to the occurrence and development of PAAD.^[35]^ Furthermore, the enriched MAPK signaling pathway is known to promote the proliferation and invasion of PAAD.^[36]^ These findings demonstrate that genomic instability-associated lncRNAs play a significant role in PAAD. In addition, GSEA suggested that high-risk patients were mainly involved with the PPP, which is closely involved in the glucose metabolism of tumor cells. During tumorigenesis and development, cancer cells need to adapt to starvation, and ATP and NADPH playing essential roles in cell growth. Hence, PPP is notably overactivated in high-risk PAAD patients, allowing cancer cells to grow in a metabolically deficient environment.^[37]^ On the other hand, low-risk patients rely on ABC transporters, which primarily act as drug transport proteins in cancer. The calcium signaling pathway has been implicated in the development of PAAD.^[38]^ Type II diabetes mellitus, as well as complement and coagulation cascades, are also play involved in tumor growth.^[39]^ In summary, genomic instability-associated lncRNAs are deeply involved in cancer development, dissemination, progression, and treatment.

By cox regression analysis, we constructed the lncRNAs signature associated with genomic instability, including potential risk prognostic lncRNAs CASC8, AC015660.1 and AC104695.4, and tumor-suppressive lncRNAs LYPLAL1-AS1, AC069120.1, AC132938.2. A few studies have shown that CASC8 promotes cell proliferation in retinoblastoma and non-small cell lung cancer.^[40,41]^ As an inflammation-related lncRNA, AC015660.1 predicts the prognosis of gastric cancer.^[42]^ AC015660.1 also can be used as an angiogenic factor-related lncRNAs to evaluate the prognosis of pancreatic cancer.^[43]^ In our study, AC015660.1, as a genomic instability-associated lncRNA, was involved in predicting the prognosis of pancreatic cancer, indicating that AC015660.1 may be a potential target for PAAD treatment. LYPLAL1-AS1 rejuvenates human adipose-derived mesenchymal stem cells and can be used as a therapeutic target to resist hADSC senescence.^[44]^ However, there is no relevant literature on its involvement in tumorigenesis. AC069120.1 and AC132938.2 were first used as prognostic factors of diseases.

To better interpret the significance of our lncRNA signature, we conducted an TME and drug sensitivity correlation analysis. TME plays an important role in the immunotherapy of tumors, and a higher immune score suggests a better therapeutic effect and survival.^[45–47]^ Currently, 3 types of immunotherapy strategies are commonly utilized in clinical settings: Checkpoint inhibitors, adoptive T-cell transfer therapy, and vaccines.^[48]^ These modalities of immunotherapy have improved outcomes for most types of tumors but did not improve the outcome of PAAD.^[49]^ The poor prognosis of PAAD can be ascribed to its biochemical complexity, including; Its pro-fibrotic, immunosuppressive, and matrix-rich tumor environment; High intra- and inter-tumor heterogeneity and; Early-stage metastasis and high resistance to chemotherapy.^[50]^ In addition to these biochemical properties, pancreatic cancer has a complex TME allowing immune escape. The complexity of the TME can be ascribed to the high infiltration of immunosuppressive cells and low infiltration of cytotoxic cells.^[51–54]^ Studies reported that resistance is caused by the poor intrinsic antigenicity of tumor cells and high abundance of regulatory T-cells in the immunosuppressive microenvironment.^[55]^ We found low immune scores in the high-risk group, while high abundances of regulatory T-cells were observed in both high-risk and low-risk groups, which can impair the efficacy of immunotherapy. We also explored potential immunotherapeutic targets for PAAD through a detailed analysis of the immune checkpoint. Results showed that the expression of all immune checkpoint-related genes was different between high- and low-risk groups. TNFSF9 and HHLA2 exhibited higher expression in the high-risk group. Wu et al^[56]^ found that overexpression of TNFSF9 was strongly associated with poor overall survival and recurrence-free survival of PAAD patients, Farrag et al^[57]^ found that HHLA2 expression is significantly correlated with metastasis of lung cancer. Approximately 83% of patients with metastasis had positive for HHLA2, compared with 44% of patients without metastasis. Our results and previous studies suggest that drugs targeting these 2 immune checkpoints may effectively for treatment of PAAD. In summary, the developed prognostic signature based on lncRNAs was found to be useful for assessing the tumor microenvironment of PAAD and can help identify potential immunotherapeutics.

Among genomic instability-associated lncRNAs, we identified 2 biomarkers, including CASC8 and LYPLAL1-AS1, for further experimental validation. CASC8, located at 8q24, is a nonprotein-coding region containing a large number of genetic loci. Studies have shown that CASC8 is associated with the occurrence and prognosis of multiple types of solid tumors, such as colorectal cancer, prostate cancer, and upper gastrointestinal cancer.^[58–60]^ Moreover, CASC8 expression was significantly different in different pancreatic cancer cell lines, with high expression in MIA PaCa-2 cells, but deficient in PANC-1 cell lines.^[61]^ Therefore, we performed further experiments to detect the expression of CASC8 in Chinese pancreatic tissues. The results showed that the expression of CASC8 in pancreatic cancer was significantly higher than that in normal pancreatic tissue. Due to our small sample size, we did not analyze the relationship between cancer grade and stage and CASC8 expression. However, the results of experimental validation were consistent with the results of the data downloaded from the database, which indicated similar pathogenesis of PAAD in Chinese patients and patients from other countries. We found that the expression of LYPLAL1-AS1 in normal tissues was significantly higher than that in tumor tissues. Currently, there is no study on LYPLAL1-AS1 and cancer, but it has been reported that LYPLAL1-AS1 is closely related to abnormal adipogenesis.^[62]^ The positive correlation between pancreatic ductal adenocarcinoma and the risk of obesity also has been confirmed.^[63]^ Therefore, it can be inferred that LYPLAL1-AS1 can affect the adipogenesis of pancreatic tissue and become a potential molecular target for PAAD. The relationship between LYPLAL1-AS1 and PAAD can be further studied.

This study also has some limitations. First, the mechanism by which genomic instability-associated lncRNAs affect the prognosis of pancreatic cancer is still unclear. Secondly, the sample size for our experimental validation is not large enough, which may affect the results. Finally, our findings need further validation through in vivo studies or clinical trials to determine their clinical applicability. Through bioinformatics analysis, we have identified potential genes that predict prognosis and guide individualized treatment. However, clinical and molecular features are highly dimensional, applying advanced machine learning methods can reduce the dimensionality of different datasets and provide more accurate signatures than traditional methods.^[64]^ This can be our next research purpose.

We constructed a prognosis-related signature for PAAD based on genomic instability-associated lncRNAs. It successfully predicted patients survival and stratified patients risk more effectively than current clinical classification methods. The signature is also useful for predicting the tumor microenvironment of PAAD and for identifying new immunotherapeutics. The prognostic biomarkers can also help to design individualized treatment strategies. We verified the expression levels of LYPLAL1-AS1 and CASC8 by in vitro experiments, which may be therapeutic targets in PAAD.

## Author contributions

**Conceptualization:** Xiuli Xia, Dongqiang Zhao.

**Data curation:** Xiuli Xia.

**Formal analysis:** Xiuli Xia.

**Methodology:** Xinying Zhu, Wenting Chen.

**Resources:** Shushan Zhao, Wenting Chen.

**Software:** Shushan Zhao, Xiaoming Song, Mengyue Zhang, Xinying Zhu, Changjuan Li.

**Validation:** Xiuli Xia, Xiaoming Song, Changjuan Li.

**Visualization:** Xiaoming Song, Mengyue Zhang.

**Writing – original draft:** Xiuli Xia, Shushan Zhao, Xinying Zhu.

**Writing – review & editing:** Changjuan Li, Dongqiang Zhao.
